# Supplementary material for: Single cell RNA-sequencing reveals that testosterone reduces estrogen signaling in the healthy human mammary gland
Source: J Mammary Gland Biol Neoplasia. 2026 Mar 25;31(1):14. doi: 10.1007/s10911-026-09605-y (PMC13132902; doi:10.1007/s10911-026-09605-y)
Supplement: Supplementary file 1 — Supplementary Material 1. SI 1: FACS sorting scheme and quality control of single cell data. a FACS gating scheme to sort for live cells from each MULTIseq-labeled sample pool. b A threshold of at least 630 UMI per cell is applied to remove empty droplets. c Cells with at most 10% of reads coming from mitochondrial genes are kept as healthy cells, while those with higher percentages are removed as unhealthy/dying cells. d UMAP embedding showing all live cells, used as input for MULTIseq barcode demultiplexing and Vireo donor classification. e Heatmap comparison of deMULTIplex2 output and Vireo output, showing agreement of most donor classifications and additional classified cells based on Vireo results. SI 2: Expression of gene markers used to annotate cell types. a UMAP embedding of demultiplexed cells with the cell type classifications. b Cell type markers used to classify each cell cluster. SI 3: Relative proportion of cells based on TRT status for each cluster and cell type, showing that some clusters are specific to cells from donors on testosterone and vice versa. SI 4: Summary of down sampling strategy for the scRNAseq dataset to balance cell type number prior to analysis with Augur. a Cell count distributions and donor-specific metrics (donor max cell count divided by mean cell count for each cell type) before and after down sampling. The process of down sampling reduces the influence of the overabundance of cells from a single donor on the Augur score. b UMAP embedding of the dataset after down sampling showed similar qualitative features as the full dataset. c Parameter sweep for the Augur algorithm showing stability of the cell type ranking across different values. SI 5: Hallmark androgen response signature in HR+ luminal cells. a Distribution of AUCell scores for the Hallmark androgen response pathway in HR+ luminal cells. b Donor average AUCell score in HR+ luminal cells for the androgen signature shows a small but significantly different androgen respo [file 10911_2026_9605_MOESM1_ESM.pdf]

Supplementary Information

Single cell RNA-sequencing reveals an association between testosterone treatment and reduced hormone signaling in the human mammary gland

Authors

Name	Affiliation
Kiet T. Phong	Department of Pharmaceutical Chemistry, University of California, San Francisco, San Francisco, CA 94158, USA
Siyou Song, MD	Department of Surgery, Division of Plastic and Reconstructive Surgery, University of California, San Francisco, San Francisco, CA, 94143, USA
Esther Kim, MD	Department of Surgery, Division of Plastic and Reconstructive Surgery, University of California, San Francisco, San Francisco, CA, 94143, USA
Danny Conrad	Department of Pharmaceutical Chemistry, University of California, San Francisco, San Francisco, CA 94158, USA
Zev Gartner, PhD*	Department of Pharmaceutical Chemistry, University of California, San Francisco, San Francisco, CA 94158, USA

* corresponding author, email address: zev.gartner@ucsf.edu

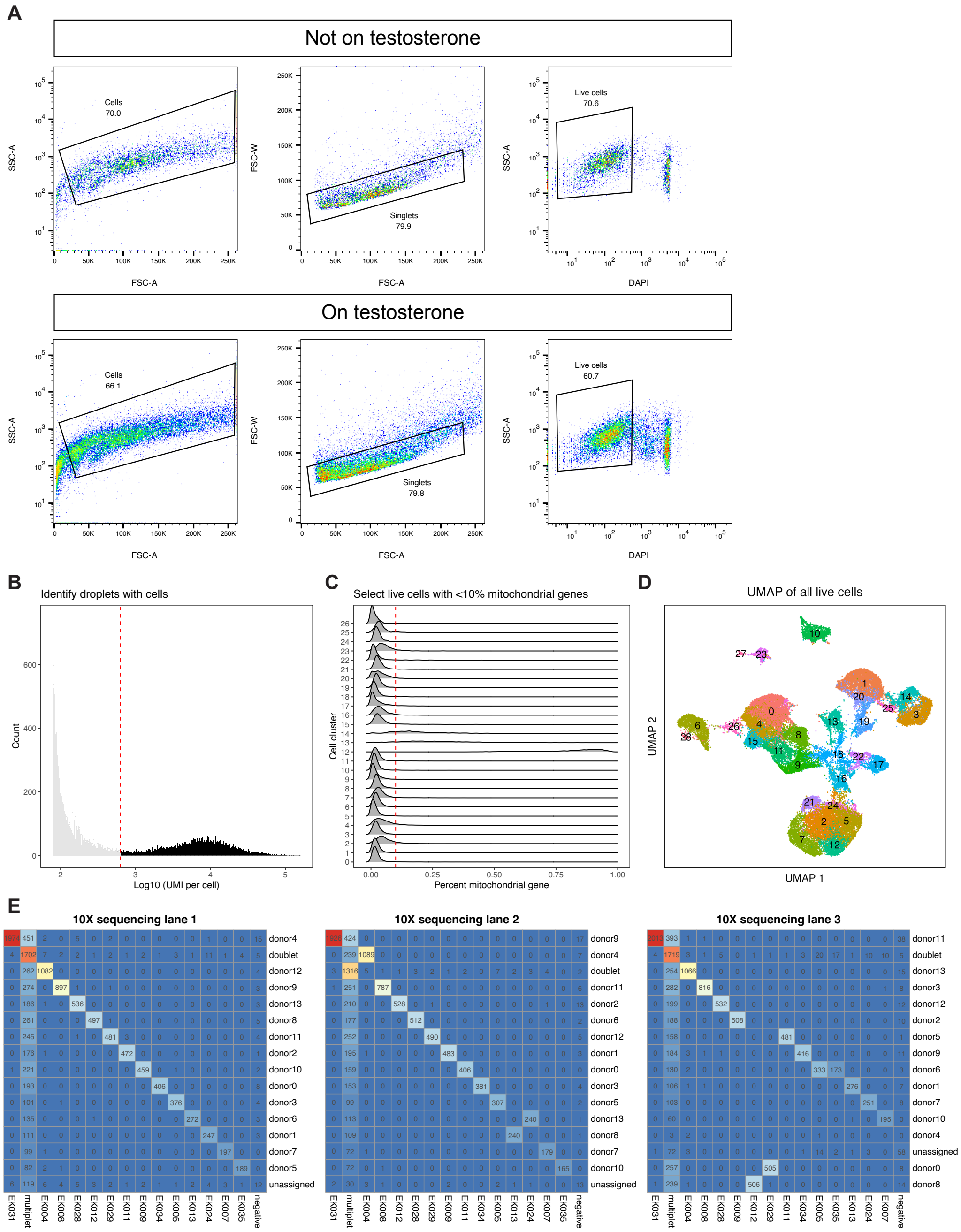

Supplementary Fig. 1 FACS sorting scheme and quality control of single-cell data

a. FACS sorting scheme to sort for live cells from each MULTIseq-labeled sample pool.
b. A threshold of at least 630 UMI per cell is applied to remove empty droplets.
c. Cells with at most 10% of reads coming from mitochondrial genes are kept as healthy cells, while those with higher percentages are removed as unhealthy/dying cells.
d. UMAP embedding showing all live cells, used as input for MULTIseq barcode demultiplexing and Vireo donor classification.
e. Heatmap comparison of deMULTIplex2 output and Vireo output, showing agreement of most donor classifications and additional classified cells based on Vireo results.

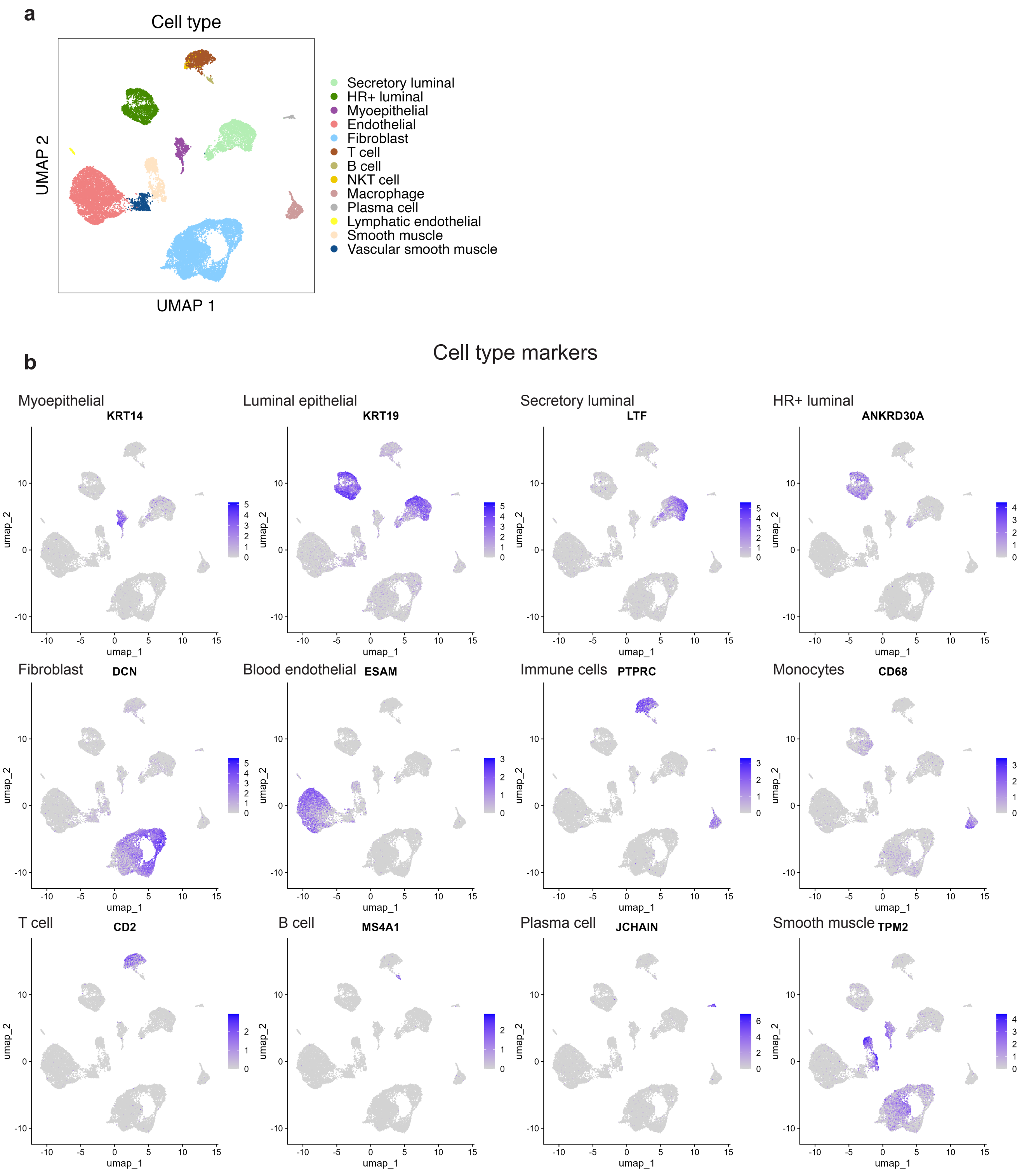

Supplementary Fig. 2 Expression of gene markers used to annotate cell types

a. UMAP embedding of demultiplexed cells with the cell type classifications.
b. Cell type markers used to classify each cell cluster.

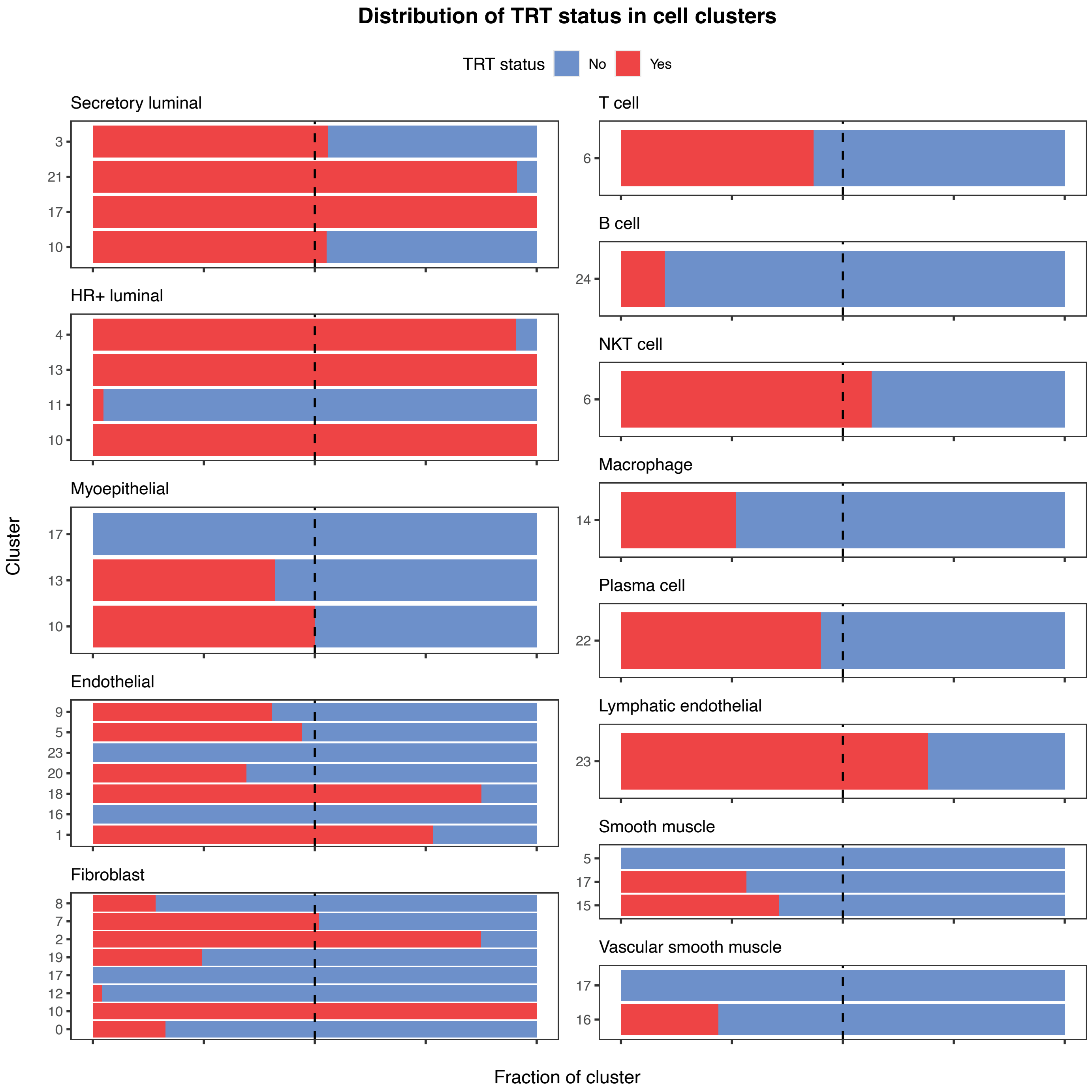

Supplementary Fig. 3 Relative proportion of cells based on TRT status for each cluster and cell type, showing that some clusters are specific to cells from donors on testosterone and vice versa.

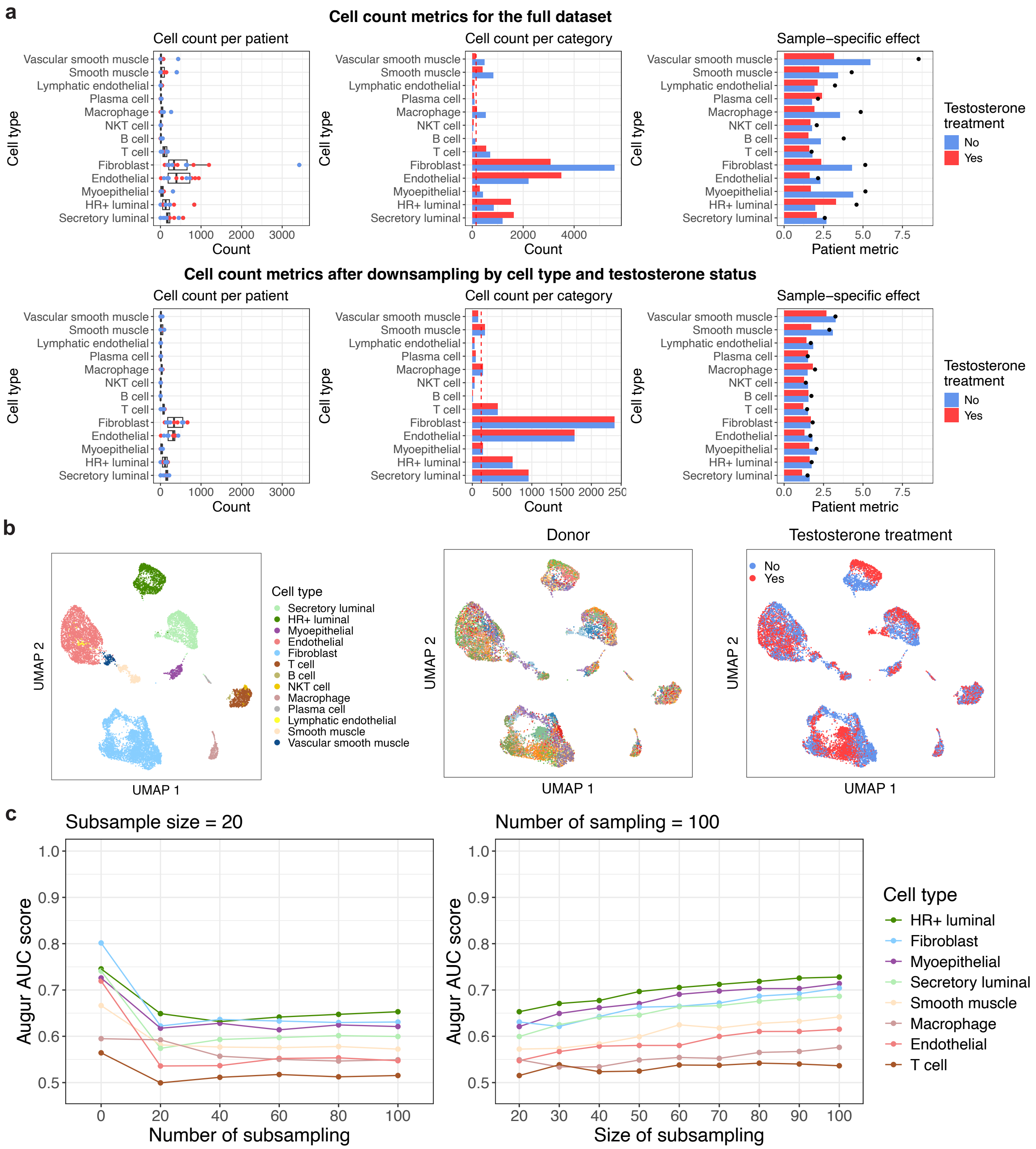

Supplementary Fig. 4 Summary of down sampling strategy for the scRNA-seq dataset to balance cell type number prior to analysis with Augur

a. Cell count distributions and patient-specific metrics (patient max cell count divided by mean cell count for each cell type) before and after down sampling. The process of down sampling reduces the influence of the overabundance of cells from a single patient on the Augur score.
b. UMAP embedding of the dataset after down sampling showed similar qualitative features as the full dataset.
c. Parameter sweep for the Augur algorithm showing stability of the cell type ranking across different values.

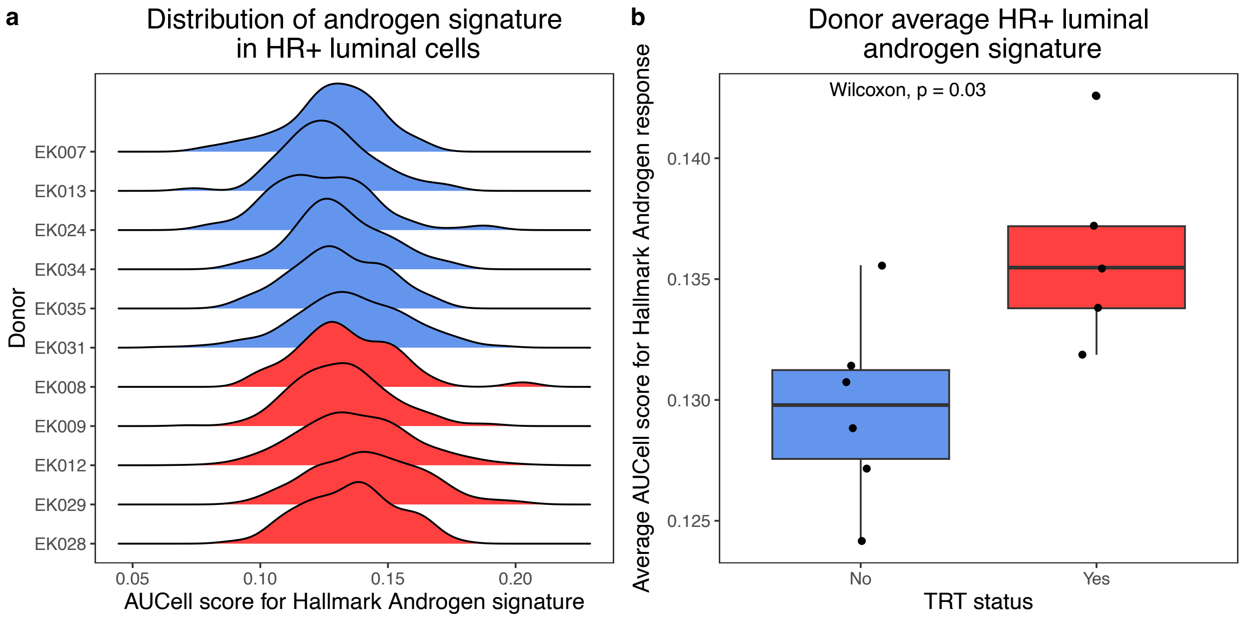

Supplementary Fig. 5 Hallmark androgen response signature in HR+ luminal cells.

a. Distribution of AUCell scores for the Hallmark androgen response pathway in HR+ luminal cells.
b. Donor average AUCell score in HR+ luminal cells for the androgen signature shows a small but significantly different androgen response in donors on TRT.

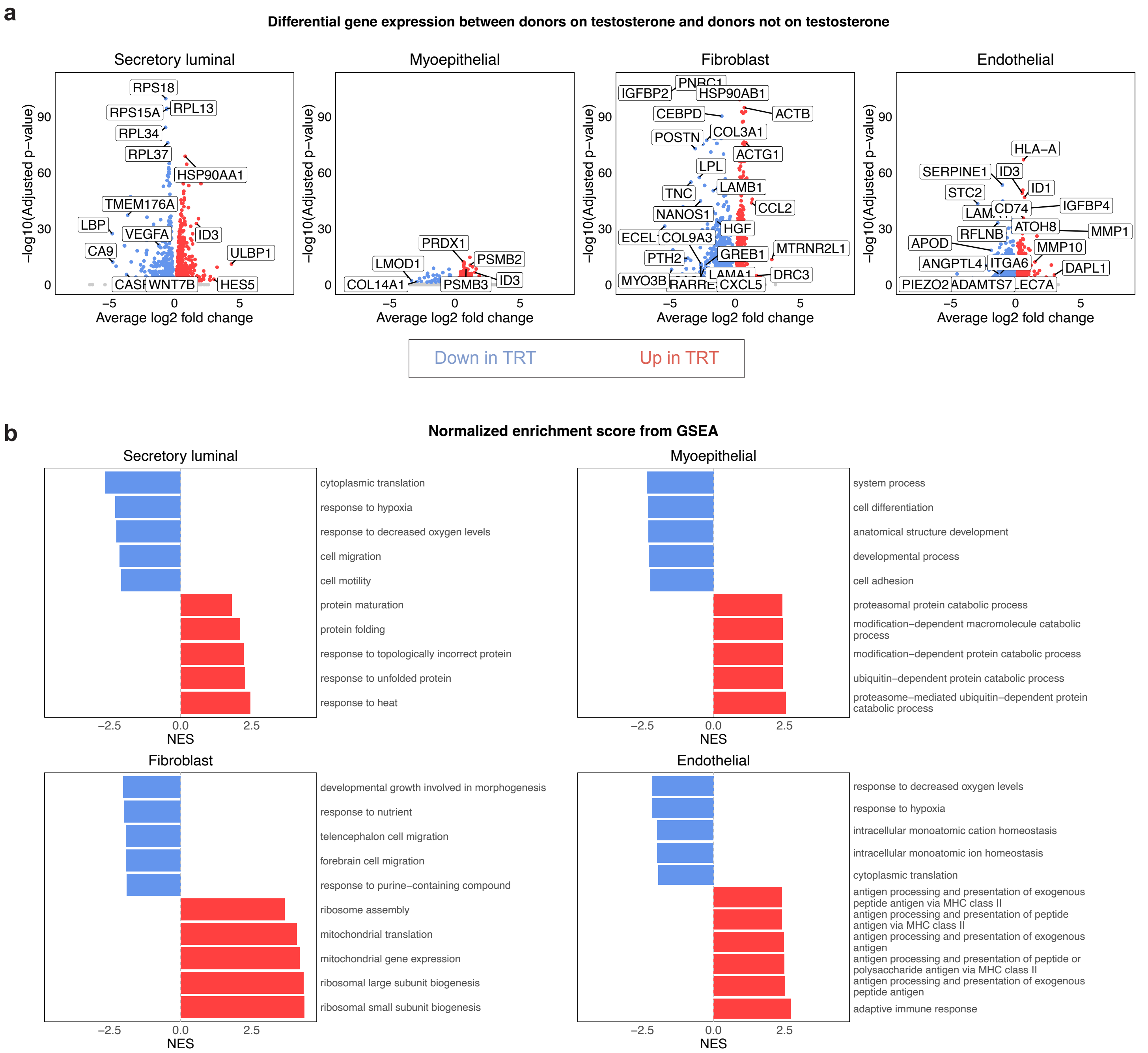

Supplementary Fig. 6 Distinct transcriptional response to TRT in the major cell types of the mammary gland.

a. Differentially expressed genes based on TRT status in each major epithelial/stromal cell type.
b. Normalized Enrichment Score of the top 10 GOBP pathways in the GSEA results based on the differentially expressed genes. A positive NES means increased enrichment in the donors on TRT, and negative score means increased enrichment in donors not on TRT.

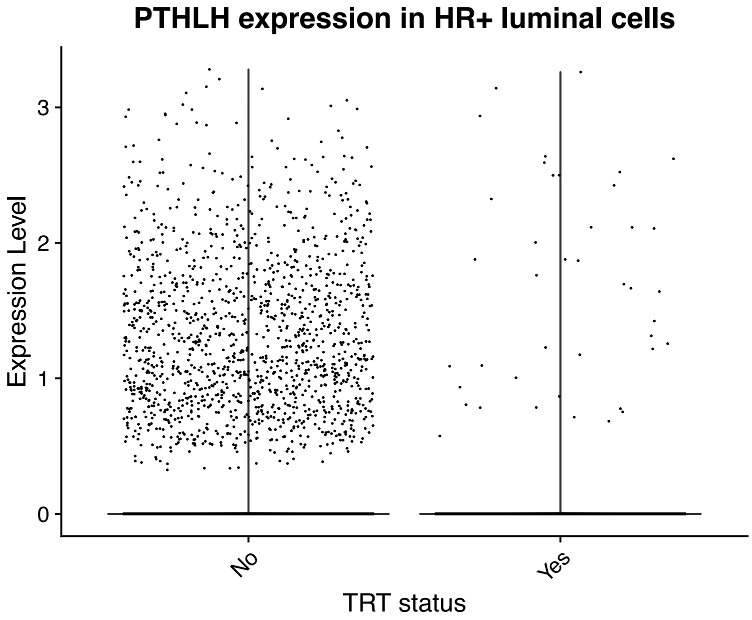

Supplementary Fig. 7 Decreased expression of PTHLH in HR+ luminal cells of participants on TRT compared to those not on TRT in the Raths et al dataset, showing agreement in the molecular finding of hormone-related signaling with the current dataset.

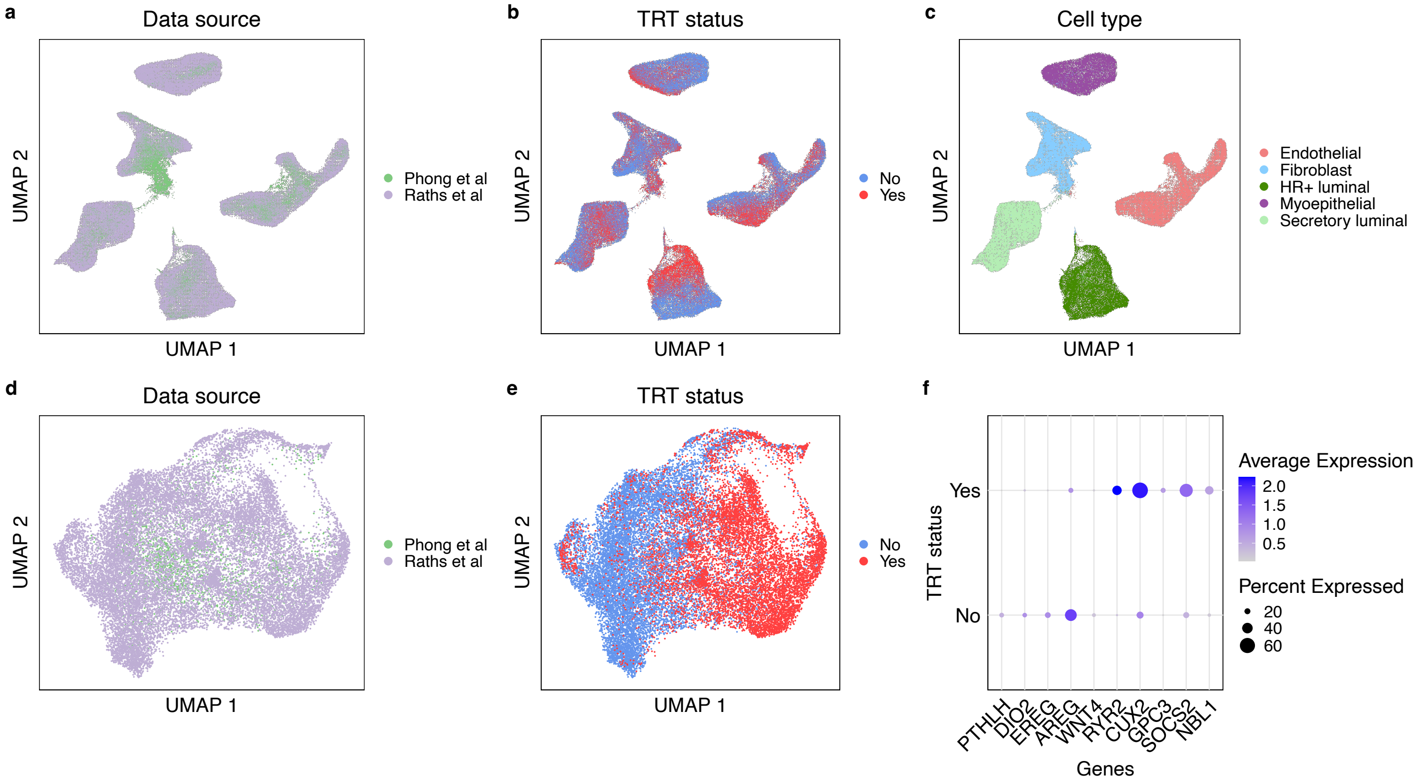

Supplementary Fig. 8 Integration of the single-nucleus RNA seq data from Raths et al and the single-cell RNAseq data from this study. We restricted the data to the three mammary epithelial subtypes, fibroblasts, and endothelial cells, then performed CCA integration to correct for batch effects and technological differences.

a. UMAP embedding of the CCA integrated dataset by data source, showing good mixing of most clusters except for the fibroblasts.
b. UMAP embedding of the CCA integrated dataset by TRT status.
c. UMAP embedding of the CCA integrated dataset by cell type.
d. UMAP embedding of the CCA integrated HR+ luminal epithelial cells by data source.
e. UMAP embedding of the CCA integrated HR+ luminal epithelial cells by TRT status.
f. Dot plot showing decreased expressions of markers of estrogen signaling (PTHLH, DIO2, EREG, AREG, WNT4) in HR+ luminal epithelial cells in donors on TRT, and increased expressions of markers downstream of androgen signaling (RYR2, CUX2, GPC3, SOCS2, NBL1).

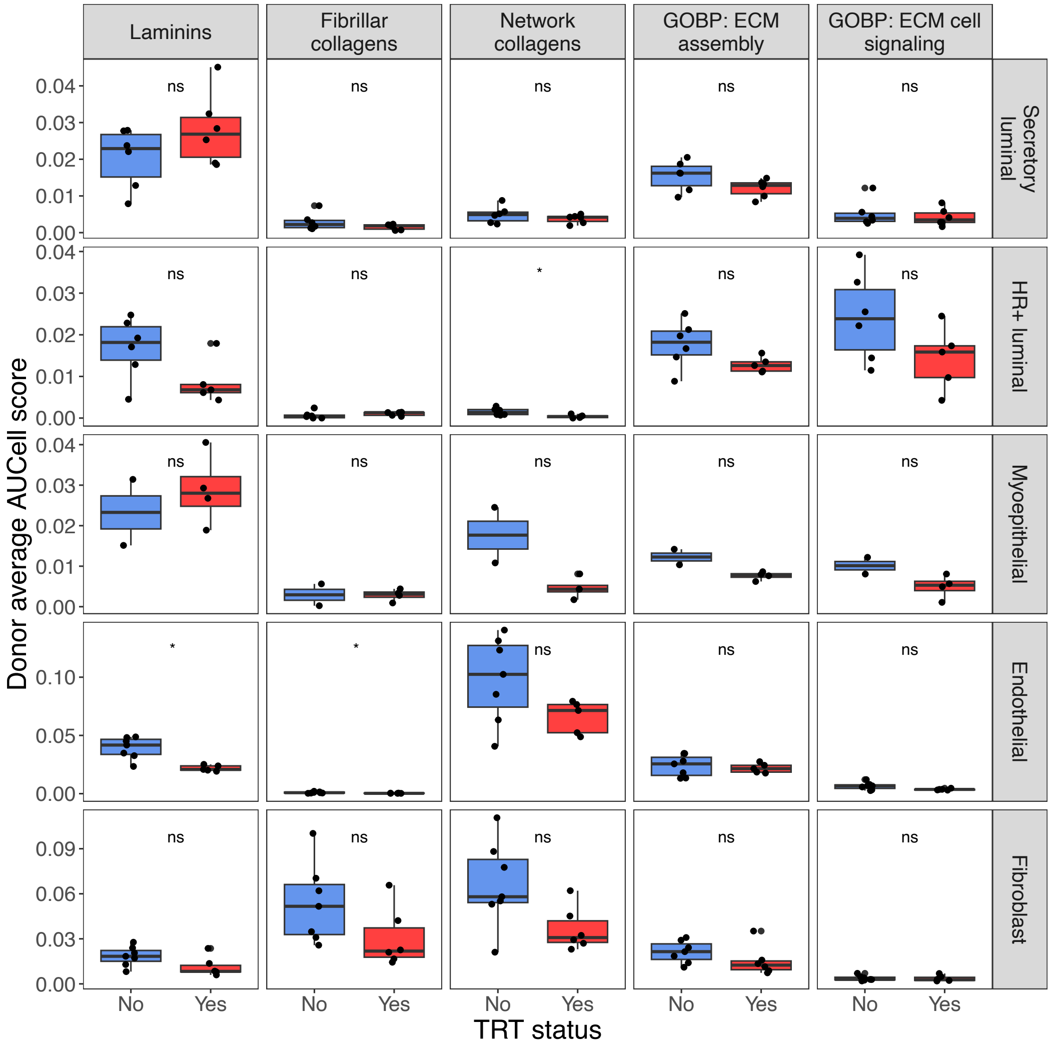

Supplementary Fig. 9 Gene expression comparison of ECM-related genes and GOBP pathways between donors on TRT and donors not on TRT in the current dataset. There is no significant difference in any of the cell types, however, there is a trend in decreased expression in donors on TRT of network collagen genes in myoepithelial cells, endothelial cells, and fibroblasts, as well as a trend for laminins, GOBP ECM assembly, and cell signaling in HR+ luminal cells. However, these results are not statistically significant, suggesting that a larger cohort in future studies should be able to resolve these differences.

Supplementary table 1: Detailed demographics information for the participants in the single cell RNA sequencing cohort

Donor	Procedure	Age	Height	Weight	BMI	Ethnicity	Regular mentrual periods? (y/n)	Age of first menstrual period	Hormone treatment history (contraceptives, etc)	Time on hormone	On testosterone?	Treatment brand	Disease history	History of breast cancer	Wt. Of tissue	Estradiol, plasma (pg/mL)	Progesterone, plasma (ng/mL)	Total testosterone, plasma (ng/dL)
EK007	Breast reduction	26	5'3"	175	31	Non-Hispanic White	Yes	12	No		No		No	Adopted	65	30	<0.1	13
EK011	Breast reduction	20	5'4"	155	26.6	Non-Hispanic White	No	13	No		No		No	No	60	28	<0.1	24
EK013	Mastectomy - gender affirming	24	6'	185	25.09	Non-Hispanic White	Yes	14	No		No		No	Paternal aunt	80	106	0.3	21
EK024	Breast reduction	19	5'4"	140	23.83	Hispanic or Latino American	Yes	12	No		No		No	Parent	42	95	7.8	11
EK031	Breast reduction	18	5'5"	185	29.95	Brazilian	Yes	10	No		No		No	No	59	104	0.1	22
EK034	Mastectomy - gender affirming	25	5'6"	127	20.35	Hispanic or Latino American	Yes	11 or 12	No		No		No	No	33	171	4.4	42
EK035	Breast reduction	24	5'4"	175	28.84	Non-Hispanic White	No	11	No		No		No	No	44	21	<0.1	31
EK004	Mastectomy - gender affirming	20	5'2"	125	23	Non-Hispanic White			testosterone	43344	Yes	testosterone cypionate (depotestosterone) 200mg/mL injection	Oligomenorrhea since age 14/15, hirsuitism, subclinical hypothyroidism August 2014. Was on Kelp pills		47	12	<0.1	384
EK005	Mastectomy - gender affirming	27	5'5"	143	23.8	Non-Hispanic White	No	15	testosterone, nexplanon	43770	Yes	testosterone cypionate, nexplanon implant	No	No	35	24	<0.1	234
EK008	Mastectomy - gender affirming	19	5'4"	150	25.8	Non-Hispanic White	No	12	testosterone	43647	Yes			No	75	67	<0.1	389
EK009	Periaeriolar mastectomy - gender affirming	21	5'10"	121	17.4	Non-Hispanic White	No	14 and 7 months	Started testosterone 2 years and 3 months ago. Started depo-provera birth control shots about 7 months ago.	Started taking 06/2019, stopped during COVID ~05/2020, resumed since then	Yes	testosterone cypionate (DEPOTESTOSTERONE) 200 mg/mL injection	No	Grandparent	40	25	<0.1	758
EK012	Mastectomy - gender affirming	31	5'8.5"	167	24.85	Hispanic or Latino American	No	9	Testosterone	Gel 08/2018, injection 03/2020	Yes	testosterone cypionate (DEPOTESTOSTERONE) 200 mg/mL injection	No	Grandparent, Parent	60	34	<0.1	618
EK028	Mastectomy - gender affirming	24	5'5"	150	24.96	Non-Hispanic White	No	13	Testosterone	1 years and 2 months	Yes	testosterone cypionate (DEPOTESTOSTERONE) 200 mg/mL injection	No	No	43	38	<0.1	507
EK029	Mastectomy - gender affirming	20	5'7"	212	33.2	Non-Hispanic White	No	12	Testosterone	43466	Yes	testosterone cypionate (DEPOTESTOSTERONE) 200 mg/mL injection	No	No	54	87	<0.1	357

Supplementary table 2: Detailed demographics information for the expanded cohort for immunofluorescence quantifications.

Sample ID	Procedure	Age	Height	Weight	BMI	Ethnicity	Regular mentrual periods? (y/n)	Age of first menstrual period	Hormone treatment history (contraceptives, etc)	Time on hormone	On testosterone ?	Treatment brand	Disease history	History of breast cancer	Wt. Of tissue	Estradiol, plasma (pg/mL)	Progesterone, plasma (ng/mL)	Total testosterone, plasma (ng/dL)
HS001	Breast reduction	26	5'5"	210		Black or African American	No	13	IUD		No	Mirena	Diabetes	No	150			
EK007	Breast reduction	26	5'3"	175	31	Non-Hispanic White	Yes	12	No		No		No	Adopted	65	30	<0.1	13
MP001	Breast reduction	21									No				70			
EK013	Mastectomy - gender affirming	24	6'	185	25.09	Non-Hispanic White	Yes	14	No		No		No	Paternal aunt	80	106	0.3	21
EK014	Breast reduction	19	5'5"	155	25.72	Non-Hispanic White	Yes	14	IUD		No	Mirena	No	Grandparent	75	71	<0.1	
EK024	Breast reduction	19	5'4"	140	23.83	Hispanic or Latino American	Yes	12	No		No		No	Parent	42	95	7.8	11
EK020	Breast reduction	18	5'6"	200	35.51	Hispanic or Latino American	Yes	13	No		No		No	No	37			
EK037	Breast reduction	22	5'1"	145	27.38	Non-Hispanic White	Yes	13	No		No		No	No	47	26	<0.1	18
EK027	Breast reduction	25	5'2"	200	36.1	Hispanic or Latino American / American Indian or Alaska Native	Yes	12	No		No		No	No	77	14	<0.1	14
EK023	Breast reduction	20	5'7"	196	30.7	Non-Hispanic White	Yes	12	progestin and estrogen		No	TRI-SPRINTEC, 28, 0.18/0.215/0.25 mg-35 mcg (28) tablet	No	No	62	<2	<0.1	6
EK035	Breast reduction	24	5'4"	175	28.84	Non-Hispanic White	No	11	No		No		No	No	44	21	<0.1	31
EK003	Mastectomy - gender affirming	39	5'7"	180	28.2	Non-Hispanic White	No	15	testosterone	42979	Yes	testosterone cypionate (DEPO-TESTOSTERONE) 100 mg/mL injection since 03/30/2020	Hypothyroidism	Grandparent, parent	130	52	<0.1	102
EK004	Mastectomy - gender affirming	20	5'2"	125	23	Non-Hispanic White			testosterone	43344	Yes	testosterone cypionate (depotestosterone) 200mg/mL injection	Oligomenorrhea since age 14/15, hirsuitism, subclinical hypothyroidism August 2014. Was on Kelp pills		47	12	<0.1	384
EK008	Mastectomy - gender affirming	19	5'4"	150	25.8	Non-Hispanic White	No	12	testosterone	43647	Yes			No	75	67	<0.1	389
EK009	Periaeriolar mastectomy - gender affirming	21	5'10"	121	17.4	Non-Hispanic White	No	14 and 7 months	Started testosterone 2 years and 3 months ago. Started depo-provera birth control shots about 7 months ago.	Started taking 06/2019, stopped during COVID ~05/2020, resumed since then	Yes	testosterone cypionate (DEPOTESTOSTERONE) 200 mg/mL injection	No	Grandparent	40	25	<0.1	758
EK021	Mastectomy - gender affirming	28	5'4"	61.7	23.4	Non-Hispanic White			Testosterone	43405	Yes	testosterone enanthate (DELATESTRYL) 200 mg/mL injection			38			
EK022	Mastectomy - gender affirming	23	5'2"	110	20.1	Everything	No	12-13	Testosterone	42917	Yes	Testosterone IM	No	No	54			
EK029	Mastectomy - gender affirming	20	5'7"	212	33.2	Non-Hispanic White	No	12	Testosterone	43466	Yes	testosterone cypionate (DEPOTESTOSTERONE) 200 mg/mL injection	No	No	54	87	<0.1	357
EK039	Mastectomy - gender affirming	22	5'6"	210	33.89	Black or African American	No		Testosterone	43983	Yes	testosterone cypionate (DEPOTESTOSTERONE) 200 mg/mL injection	No	No	41	88	<0.1	1210

Supplementary table 3: The molecular changes in each cell type in transgender men on testosterone replacement therapy (TRT) relative to cisgender women in the current study, in comparison to a previous study. Data for previous study is derived from Raths F, Karimzadeh M, Ing N, et al (2023) The molecular consequences of androgen activity in the human breast. Cell Genom 3:100272. https://doi.org/10.1016/j.xgen.2023.100272

Green: agreement between the datasets
Red: disagreement between the datasets

Cell type	Pathway / Gene	Direction of change in transgender men in Raths et al.*	Direction of change in transgender men in this dataset
**Secretory luminal**	ITGA	Down	ITGA6 up, others not significant
	ITGB	Down	ITGB1, 2, and 4 are down, but p-value not significant
	AZGP1	Down	Not significant
	KEGG: focal adhesion	Down	Down
	KEGG: adherens junctions	Down	Down
	KEGG: actin cytoskeleton regulation	Down	Not significant
**HR+ luminal**	WP: fatty acid biosynthesis	Up	Up
	KEGG: calcium signaling pathway	Up	Down
	BIOC: igf1 pathway,	Down	Down
	WP: mammary gland development pathway	Down	Down
	HALLMARK: estrogen response early	Down	Down
	PGR	Down	Down
	AR	No difference	No difference
	ESR1	No difference	No difference
	JUN	Down	Down
	AREG	Down	Down
	EREG	Down	Down
	ADAM17	Down	Down
**Myoepithelial**	ITGB1	Down	Not significant down
	ACTA2	Down	Down
	OXTR	Down	Not significant down
	TP63	Down	Not significant down
	PROS1	Down	No difference
	BACH2	Down	No difference
	REAC: smooth muscle contraction	Down	Down
	KEGG: focal adhesion	Down	Down
	REAC: cell junction organization	Down	Down
**Fibroblast**	LAMB1	Down	Down
	LAMA2	Down	Down
	FN1	Down	Down
	THBS1	Up	Up
	IL16	Up (in 1 subset)	No difference
**Vasculature**	Lymphatic & capillaries	Lower	Not enough cells
	PPARG	Down	Down
	PPARG RGN	Down	KEGG pathway unclear
	VEGFR2	Up	Slight up, not significant
	FLT4	Up	Low expression, slight up, not significant
**Immune**	Macrophage count	Down	Not enough cells
	Macrophage REAC: class I MHC mediated antigen	Down	No difference
	Macrophage REAC: clathrin-mediated endocytosis	Down	Slightly down
	Macrophage REAC: TLR1 TLR2 cascade	Down	Slightly down
	CD4 T cells	Up	Not enough cells
